# Croton Tiglium

**Published:** 1888-06

**Authors:** J. H. Hamer


### CROTON TIGLIUM.

(Read before Hahnemannian Association of Pennsylvania.)

Croton oil is expressed from the seeds of the croton tiglium, a plant which belongs to the family of the Euphorbiaciæ. In the same family is found the Ricinus coni., from which is prepared the Castor oil with which, doubtless, many of us have made unpleasant acquaintance in the course of practice. Given in crude doses of from one to ten gtt. internally it produces violent purging. In large doses it produces violent vomiting, collapse, and death, the symptoms strongly resembling those of Asiatic cholera, or, if the patient survives, he suffers from chronic gastro-enteritis. Its local action upon application to the skin speedily produces a copious eruption of pustules, accompanied with great pain and burning. Epigastric pain, soreness of the eyes, and swelling of the face have been observed to follow the inhalation of the dust in those who have to do much handling of the seeds.

Its range of action as a therapeutic agent is rather limited, being confined mostly to affections of the skin and mucous membranes, particularly to the mucous membrane of the intestinal tract. It has consequently proved particularly effective, when indicated, in diseases which manifest their morbific influence most distinctly upon the intestinal tract, *e. g.*, cholera infantum, cholera, diarrhœa, dysentery etc., or upon the skin when accompanied by the characteristic indications which call for this remedy, *e. g.*, crusta lactea, ecthyma, eczema, erythema, herpes, impetigo, itch, pruritis, erysipelas, Rhus poisoning, etc., especially in diseases which take on a vesicular or pustular form. It is also useful when indicated by its characteristic symptom, in diseases of the mucous membrane elsewhere than in the intestinal tract, *e. g.*, blepharitis, keratitis, ulceration of the cornea ophthalmia (glaucoma) in the eye, cough in the respiratory tract, prostatitis in the urinary passages. In irritable and cracked nipples during lactation, in mastitis, and sometimes in headache or neuralgia when called for it proves effective.

Following are the symptoms in detail :

### Head.

Vertigo, with dullness and heaviness of the head, paleness, debility and nausea, worse in open air and in the morning, sensitiveness to pressure of the hat. In the characteristic headache the pains extend backward from the eyeballs through the head, or are located in the superciliary region over right eye.

### Eyes.

Itching, œdema, twitching of lids, stinging in eyeball, burning in eye and ear with vertigo and fainting, ulceration of conjunctiva, contraction of pupil, dimness of cornea, lachrymation, neuralgic pains from pupil of left eye through to back part of head.

The characteristic eye troubles, conjunctivitis, blepharitis, ulceration of cornea, etc., are accompanied by vesicular eruptions on adjacent parts and characteristic pain shooting from the eye to back of head, and the corneal ulceration by marked pain at night in the superciliary region.

### Nose.

Burning in nares and inflammation of the nose.

### Mouth and Throat.

Dry, parched lips, burning of lips, sensitiveness of tip of tongue, coated white, disagreeable scraping in fauces, burning in fauces and pharynx, sensation of constriction in throat.

### Stomach.

Excessive nausea with vanishing of sight and sweat on forehead, excessive gagging with vertigo, these symptoms worse after drinking, burning in stomach with sensitiveness to touch.

### Abdomen.

Distended, coldness, colic in transverse colon before stool, relieved by drinking hot milk and after sleep.

### Stool.

Liquid yellow or dark green with tenesmus, nausea and colic, coming out with a spurt as from explosive spasm. Stools immediately after drinking or eating. In infants immediately or soon after nursing. Burning at anus, itching, pulsative stitches, sense of constriction, as if a plug lodged in rectum were pressing out, scraping on posterior wall of rectum during stool, pressure at umbilicus causes pain all along the canal to termination of rectum, producing tenesmus and protrusion of rectum, sweat during stool and warm sweat on forehead after stool.

In diarrhœa, cholera, cholera infantum, and cholera morbus, the three prominent and highly characteristic indications for the exhibition of Croton tiglium are the yellow, watery stools, the sudden expulsion, and the aggravation from eating or drinking.

The attacks are marked by violent nausea and vomiting of the ingesta, sudden attacks of vomiting of yellowish white, frothy fluid, with violent efforts of the stomach to dislodge it, accompanied by anguish, oppression, burning, and pressure in stomach; much water in the mouth; violent purging with tenesmus and disagreeable sensation through the whole body and nauseous taste; dry, parched lips; blind vertigo; colic; sweat during stool, followed by excessive prostration and fainting.

Stools may resemble gray mucus, and are marked by great debility. The diarrhœa may be intermittent with great and sudden weakness or morning diarrhœa, light, watery, almost painless, followed by prostration.

In dysentery the stools are frequent and small, renewed by every movement of the body, with very violent pain in the bowels at first, with tenesmus.

### Urinary Organs.

Stitches in region of kidneys arresting breathing; increased flow of turbid urine; burning in urethra on urinating; urine, greasy, with opalescent pellicle on surface; desire to urinate continues after micturition.

### Sexual Organs.

*Men.*—Left testicle drawn up; right relaxed.

*Women.*—Menses scanty during confinement and lactation; nipples very sore to touch; excruciating pain running from the nipple through the chest to scapula of same side as soon as the child begins to nurse; irritated nipples from deficiency of milk.

This pain from the nipple through the chest is a loud call for a dose of Croton tiglium.

### Respiratory Organs.

Hoarseness, hollow voice, constant hawking, accumulation of rattling mucus in larynx; cough morning and evening, with soreness in abdomen and burning in chest; accumulation of rattling mucus in chest, which is painful to the touch. Cough, with violent sore, drawing pain through chest to back, more on left side; sense of hollowness in chest.

### Heart.

Palpitation, with difficulty of breathing, especially upon going up stairs; stitches in region of heart during expiration; drowsiness, but palpitation of heart prevents sleep.

### Extremities.

Sticking or aching pains in the shoulder joints. Lancination in great toes; pricking in toes.

### Fever.

Pulse frequent and full; chilliness over the back; heat ascends over the body; perspiration on the forehead.

### Skin.

Scarlet redness of skin, with rashlike vesicles; redness, warmth, stinging here and there; pustules running into one another, oozing and forming a gray-brown crust; itching, followed by painful burning; herpetic eruption on scrotum; painful swelling of submaxillary glands and tonsils; eruptions better by gentle rubbing.

The characteristic eruption of Croton tiglium is pustular, or seropurulent, marked by intense *burning* and itching, ameliorated by gentle rubbing or scratching and after sleep.

In crusta lactea, herpes, impetigo, ecthyma, eczema, erythema, hydroa, itch, poisoning by Rhus tox., pruritis, and erysipelas, or any other skin disease, when characterized by this intense burning and itching, which finds relief from gentle friction, you may safely prescribe Croton tiglium.

### Generalities.

Weariness, ill-feeling, and irresistible drowsiness, fainting spells, weakness, and bruised feeling through the body.

### Conditions.

*Agg.*—Stools, nausea, vomiting, and colic after eating or *drinking.*

*Amel.*—Headache, colic, and pains after sleeping, eruptions from gentle friction.

J. H. Hamer, M. D.

